# Argyrol in Ulcerative Colitis; the Vomiting of Enemata

**Published:** 1904-10-01

**Authors:** 


### ARGYROL IN ULCERATIVE COLITIS; THE VOMITING OF ENEMATA.

In recording a case of ulcerative colitis Dr. W. Murrell[1] draws attention to the good effect following the injection of a solution of argyrol into the bowel. The regulation of the diet and the use of lead acetate and other astringents, had been practised without avail before argyrol was tried. The plan adopted was as follows:—The patient was first given an enema of boric acid, 8 grains to the pint, and then by an irrigation tube of small diameter a 1 per cent. aqueous solution of argyrol at 80° was used. About 5 pints were introduced, the patient being in the knee and elbow position. The frequency of the motions promptly diminished, and the temperature rapidly fell to normal. As a matter of precaution the treatment was repeated on two subsequent occasions. But there was no return of the diarrhœa, and in the course of a few days meat, potatoes, and green vegetables could be taken with impunity; the patient began to gain weight, and ultimately he made an excellent recovery.

A fact of much interest was noted in the case—namely, that during the rectal administration of the argyrol solution a claret-coloured fluid identical in appearance with that injected escaped from the mouth, and that, too, without retching or vomiting. This, it was at first suggested, was altered blood or bile, but when analysed by Dr. Wilson Hake was proved to contain silver, and not to differ materially from the 1 per cent. argyrol solution. There was not the slightest reason for believing that such an unusual condition as the existence of a short circuit between the rectum and the stomach existed, and Dr. Murrell regards the event as fairly comparable to the vomiting of enemata by hysterical and other patients. Of such instances he quotes a considerable number recorded in medical literature. Among these is a case reported by Sennest, in which a girl of 12 years vomited a large suppository after introduction into the rectum "dans le temps de réciter un *Pater noster* et un *Ave Maria*." A second suppository attached to a thread, and a third "secured by four strong ligatures" were treated in the same fashion.

There have been numerous examples of the vomiting of enemata. In one case, recorded by Dr. Parkes Weber,[2] when an enema of oil was given the oil reappeared in the vomited matter, and the same was true of a solution of methyl blue. To exclude the possibility of a gastro-colic or duodeno-colic fistula the stomach was emptied by the syphon-tube immediately after the administration of the enema, and in these circumstances none of the drug was found in the stomach contents, showing that it took at least some minutes to reach the stomach. And all possible doubt on the matter is removed by the fact that the patient had on two occasions been submitted to laparotomy without the discovery of anything abnormal. In the light of these and other similar occurrences it is open to doubt whether the ileo-cæcal valve is so perfect a mechanism as the anatomists suggest, and as seems to be demonstrated by the ordinary dissecting-room test. Fæcal vomiting, apart from the use of enemata, also seems to be a well-established fact. In a case described by Jaccoud (quoted in Dr. Murrell's article) a hysterical woman vomited formed motions, cylindrical in shape, brown in colour, and possessing a characteristic fœcal odour. Bregman[3] describes a similar occurrence, and on several occasions actually witnessed the vomiting of fæces. Another of the more unusual phenomena of hysteria is anuria, an example of which is reported by Scarpini.[4] The patient, when 19 years of age suffered from dysuria needing constant catheterism for three months. At 2[illegible] she had a sudden hæmaturia without apparent cause. Later she developed hemiplegia which lasted almost eight months and after this anuria was observed. This was associated with œdema of the lower limbs, nausea and headache. Vomiting after food became a constant feature and the vomit had the character and appearance of urine, the quantity being about 400 grams daily. The anuria was practically complete though occasionally a few drops of urine could be withdrawn by the catheter; it did not contain albumen. The bowels were opened slightly once every 15 days or so. There were various disturbances of sensation, but the tendon jerks were normal and no evidence of organic disease could be discovered. Altogether the anuria lasted about two years. Charcot[5] described a case in which no urine was passed for 11 days, and during this time the patient vomited feeely and the vomit contained urea in considerable amount; the natural flow of urine returned spontaneously and the patient made a complete recovery.

[1] Lancet, August 13, 1904. [2] St. Bartholomew's Hospital Reports, 1898. [3] Med. Rev., December 1901. [4] Brit. Med. Journ. (Epitome), August 13, 1904. [5] Diseases of the Nervous System, vol. i. (New Sydenham Soc.).

